# The vascular microenvironment and its stem cells regulate vascular homeostasis

**DOI:** 10.3389/fcell.2025.1544129

**Published:** 2025-03-06

**Authors:** Yanhui Wang, Xiaoyun Zhang, Xin Li, Min Cheng, Xiaodong Cui

**Affiliations:** Medical Physiology Laboratory, School of Basic Medical Sciences, Shandong Second Medical University, Weifang, China

**Keywords:** vascular microenvironment, stem cells, endothelial progenitor cells, mesenchymal stem cell, vascular homeostasis

## Abstract

The vascular microenvironment comprises of anatomical structures, extracellular matrix components, and various cell populations, which play a crucial role in regulating vascular homeostasis and influencing vascular structure and function. Under physiological conditions, intrinsic regulation of the vascular microenvironment is required to sustain vascular homeostasis. In contrast, under pathological conditions, alterations to this microenvironment lead to vascular injury and pathological remodeling. According to the anatomy, the vascular microenvironment can be subdivided into three sections from the inside out. The vascular endothelial microenvironment, centered on vascular endothelial cells (VECs), includes the extracellular matrix and various vascular physicochemical factors. The VECs interact with vascular physicochemical factors to regulate the function of various parenchymal cells, including hepatocytes, neurons and tumor cells. The vascular wall microenvironment, comprising the vasa vasorum and their unique stem/progenitor cell niches, plays a pivotal role in vascular inflammation and pathological remodeling. Additionally, the perivascular microenvironment, which includes perivascular adipose tissue, consists of adipocytes and stem cells, which contribute to the pathological processes of atherosclerosis. It is anticipated that targeted regulation of the vascular microenvironment will emerge as a novel approach for the treatment of various diseases. Accordingly, this review will examine the structure of the vascular microenvironment, the regulation of vascular function by vascular cells and stem/progenitor cells, and the role of the vascular microenvironment in regulating cardiovascular diseases.

## 1 Introduction

Homeostasis refers to a state in which an individual organism maintains a relatively stable internal environment, characterized by a balanced and harmonious state at all levels of life activity, including cells, tissues, organs, and the whole organism (Meizlish et al., 2021). Vascular homeostasis involves multiple aspects, which include alteration and remodeling of vascular function, vascular injury and repair, as well as vascular neogenesis and angiogenesis (Zhang and Dong, 2014). Therefore, understanding the regulatory phenomena and underlying mechanisms of vascular homeostasis holds significant research value and practical importance for the modulation of vascular function.

Vasculopathy is suggested to be caused by dysregulation of the vascular niche, a microenvironment within the vascular structures that includes anatomical components, extracellular matrix elements, and various cell populations (Dergilev et al., 2024). This vascular niche, also known as the vascular microenvironment, plays a critical role in homeostatic regulation to maintain tissue, organ function, and overall biological activity. Under physiological conditions, the vascular microenvironment is finely regulated to maintain the normal structure and function of the vasculature (Nikolova et al., 2007). Conversely, under pathological conditions, vascular cells are affected by physicochemical vascular microenvironmental factors. As a result, they undergo functional changes leading to vascular injury and remodeling (Lei et al., 2022). Such alterations in vascular function can significantly affect the performance of the tissues and organs they service. Furthermore, vascular cells can secrete cytokines to regulate the function of neighboring cells (Chen and Ding, 2022). Therefore, modulation of the vascular microenvironment is a promising research avenue to influence tissue and organ function.

The arterial vessel wall is structurally divided into three main layers: the tunica intima, tunica media and tunica adventitia. The intima comprises vascular endothelial cells (VECs) and the vascular basement membrane. The tunica media predominantly includes smooth muscle cells (SMCs) (Seidelmann et al., 2014), while the tunica adventitia consists of connective tissue and fibroblasts. In addition, the vascular wall contains the vasa vasorum (VV), which are responsible for suppling nutrients to the walls of arteries and veins (Mulligan-Kehoe and Simons, 2014). Surrounding the vascular wall is perivascular adipose tissue (PVAT), which envelops systemic blood vessels except in the case of cerebral vessels (Chang et al., 2020). PVAT is a specialized form of adipose tissue located adjacent to the outer layer of blood vessels and is primarily composed of adipocytes, fibroblasts, stem cells, mast cells, and neuronal cells (Hillock-Watling and Gotlieb, 2022).

In addition to VECs, SMCs, fibroblasts, and macrophages, the tunica adventitia and PVAT also harbor resident stem and progenitor cells, which play critical roles in the processes of vascular inflammation, repair, and remodeling (Tinajero and Gotlieb, 2020). These stem/progenitor cells include mesenchymal stem cells (MSCs), smooth muscle progenitor cells, and endothelial progenitor cells (EPCs), all of which possess multidirectional differentiation potential and regenerative capabilities (Rohban et al., 2017; Steens et al., 2021). Consequently, these cells are integral to the vascular microenvironment, contributing significantly to its regulation and function.

This article seeks to provide a comprehensive overview of the vascular microenvironment, highlighting the pivotal role of stem/progenitor cells in maintaining vascular health and responding to pathological changes.

## 2 Concept of vascular microenvironment

**FIGURE 1:**
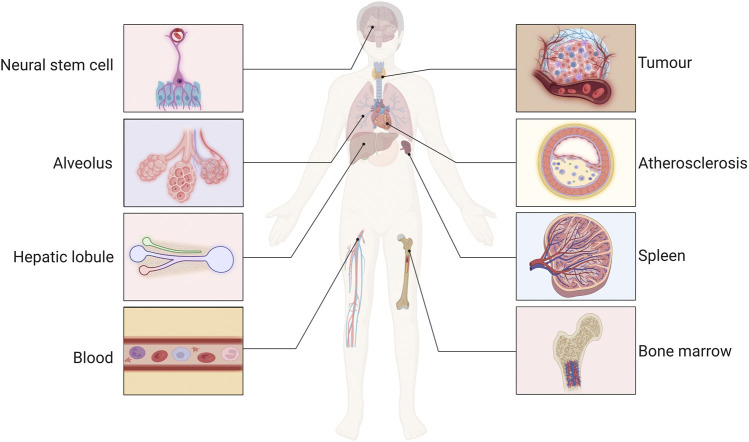
The function of the vascular microenvironment. The maintenance, activation and remodeling of the vascular microenvironment regulate the function of neurons, immune, tumor, liver, and spleen cells, and involved in the processes of bone marrow hematopoietic activity, liver regeneration and repair, regulation of neural stem cell function, atherosclerotic (AS) inflammatory response, and pathological vascular remodeling.

The vascular microenvironment can be conceptualized as a dynamic microecosystem that consists of VECs residing in the intima, SMCs located in the subendothelial space, and connective tissue cells, such as fibroblasts and macrophages. These cellular components engage in complex interactions via ligand–receptor signaling, exosome exchange, and cytokine communication, collectively regulating vascular tone and maintaining cellular homeostasis (Xu et al., 2022). Cells within the vascular microenvironment of various tissues respond to a range of physiological and pathophysiological factors, which regulate cellular functions, including growth, differentiation and transformation of specific cells, such as neurons (Sbierski-Kind et al., 2021), arterial VECs (Mao et al., 2020), and immune cells (Li et al., 2018; Ji et al., 2021). Additionally, the vascular microenvironment influences the hematopoietic activity and osteogenesis of the bone marrow (BM) (Chi et al., 2021; Yu et al., 2022) and plays a role in the regenerative repair processes within liver parenchymal cells (Duarte et al., 2018). Furthermore, the vascular microenvironment in the lung alveoli may play an important role in regenerative tissue repair, senescence and fibrosis (Kato et al., 2018; Chen J. et al., 2020; Gomez-Salinero et al., 2021; Termini et al., 2021; Yan et al., 2021). Modifications to the tumor vascular microenvironment have also been demonstrated to influence the stemness and invasiveness of tumor cells. Furthermore, alterations in the vascular microenvironment contribute to the development of atherosclerosis (AS) by promoting inflammatory responses and vascular remodeling (Lu et al., 2020). Therefore Poulos et al. (Poulos et al., 2014) proposed the development of drugs that target the vascular microenvironment as a new therapeutic tool (Figure 1).

Increasing evidence suggests that targeting the vascular microenvironment plays a critical role in regulating tissue cell function. In murine models, overexpression of hepatocyte growth factor (HGF) and inhibition of profibrotic NADPH oxidase 4 (NOX4) in VECs creates a modified vascular microenvironment in the liver and lung tissue that promotes liver and lung fibrosis. Attempts to intervene in this nascent microenvironment promotes the regeneration of fibrotic organs (Cao et al., 2017). In addition, Cao et al. (2016) injected bleomycin or hydrochloric acid into the lungs of mice to replicate a lung injury model and found that capillary endothelial cells, macrophages, and fibroblasts pathologically reconstituted the vascular microenvironment in the lungs, which impaired lung tissue repair. Therefore, studying the vascular microenvironment is novel direction disease therapeutics.

**FIGURE 2:**
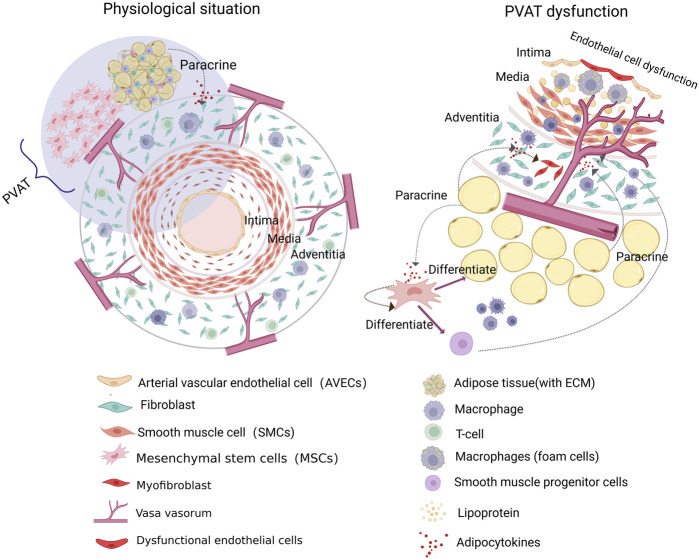
The composition of the vascular microenvironment. The vascular microenvironment can be subdivided into three sections according to the vascular anatomy, from the inside out. The vascular endothelial microenvironment is centered on the vascular endothelial cells (VECs) and encompasses the extracellular matrix and vascular physicochemical factors. The vascular wall microenvironment, comprising the vasa vasorum (VV) and their distinctive stem/progenitor cell niches, represents a pivotal site implicated in vascular inflammation and pathological remodeling. Additionally, the perivascular microenvironment, comprising perivascular adipose tissue (PVAT), contains adipocytes and stem cells that may contribute to the pathological process of atherosclerosis (AS).

To better understand the vascular microenvironment, we divided the complex network into three parts based on the vascular anatomy from the inside out: the vascular endothelial microenvironment, vascular wall microenvironment, and perivascular microenvironment. The vascular endothelial microenvironment is focus on the VECs (Nikolova et al., 2007; Manavski et al., 2014). The vascular wall microenvironment is characterized by the VV, whose unique stem/progenitor cell niches play a crucial role in monitoring, maintaining, renewing, and replenishing key elements of the vascular endothelial microenvironment (Zaniboni et al., 2015). Finally, the perivascular microenvironment, which includes PVAT, consists of stem/progenitor cells and adipocytes that influence vascular pathophysiology (Ozen et al., 2015; Nosalski and Guzik, 2017) (Figure 2). Regulation of the vascular microenvironment can significantly alter the functional state of tissue cells and influence disease progression. Understanding these mechanisms is crucial for identifying potential molecular targets within the vascular microenvironment for therapeutic intervention.

## 3 The vascular endothelial microenvironment and related stem cells

### 3.1 The VECs influence the function of their surrounding cells

In 2014, Géraud et al. (2014) highlighted the critical role of VECs within the vascular microenvironment. VECs serve as a selective diffusion barrier between the blood and arterial wall, making them particularly sensitive to changes induced by biochemical and fluid factors in the bloodstream (Milutinović et al., 2019). The physicochemical factors present in the vascular microenvironment directly influence the function of VECs, which then regulate the activity of surrounding cells through cytokine secretion. For instance, VECs secrete signaling molecules that regulate thrombosis by modulating vascular tone and preventing platelet and leukocyte adhesion (Chi et al., 2022). In this context, we summarize the role of VECs and vascular physicochemical factors as key components of the vascular endothelial microenvironment. We also discuss the inter-regulatory interactions between VECs and physicochemical factors and highlight the relationship between stem cells and the vascular endothelial microenvironment in different tissues.

#### 3.1.1 Hepatic vascular microenvironment

**FIGURE 3:**
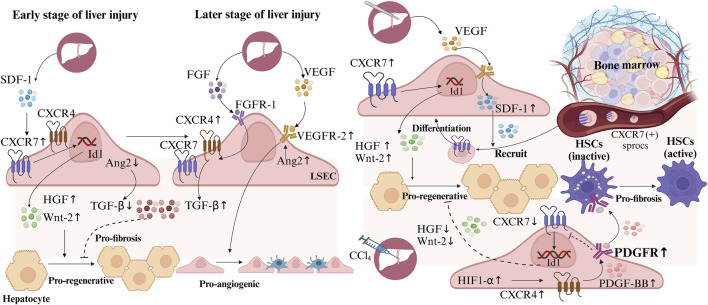
Liver sinusoidal endothelial cells (LSECs) in hepatic regeneration and fibrosis following liver Injury. During early liver injury, upregulated CXC-chemokine receptor 7 (CXCR7) and downregulated Ang2 in LSECs, increase hepatocyte growth factor (HGF) and Wnt-2, reduce transforming growth factor-β (TGF-β), creating a pro-regenerative microenvironment. Later, Ang2 upregulation enhances vascular endothelial growth factor receptor-2 (VEGFR-2), forming a pro-angiogenic microenvironment. Persistent fibroblast growth factor receptor 1 (FGFR1) signaling boosts CXCR4 and TGF-β, inducing a pro-fibrotic microenvironment. After hepatectomy, LSECs secrete stromal cell-derived factor 1 (SDF1) to recruit bone marrow CXCR7 (+) progenitors of liver sinusoidal endothelial cells (sprocs), which differentiate into LSECs. In CCl_4_-induced liver injury, hypoxia-inducible factor-1α (HIF-1α) elevates CXCR4 and platelet-derived growth factor-BB (PDGF-BB), inhibits CXCR7, while PDGF-BB activates hepatic stellate cells (HSCs), promoting a pro-fibrotic microenvironment.

VECs secrete various growth factors, collectively known as angiopoietins (Ang), which influence the biological functions of VECs and surrounding cells. Ang2 plays a critical role in regulating hepatocyte growth, development, regeneration, and neural stem cell function (Androutsellis-Theotokis et al., 2010; Hong et al., 2021). In the hepatic vascular microenvironment, liver sinusoidal endothelial cells (LSECs) exert paracrine regulatory effects on hepatocytes and hepatic stellate cells (HSCs) (DeLeve, 2015). Following liver injury, precise temporal and spatial regulation of CXC-chemokine receptor 4/7 (CXCR4/7) and Ang2 expression in LSECs is crucial for liver reconstitution (Manavski et al., 2014). In the early stages, upregulation of CXCR7 and downregulation of Ang2 in LSECs promote HGF and Wnt-2 expression, while inhibiting transforming growth factor-β (TGF-β) production, creating a pro-regenerative microenvironment that supports liver regeneration and repair (Ding et al., 2014; Hu et al., 2014). In contrast, during later stages of injury, sustained signaling through fibroblast growth factor receptor 1 (FGFR1) in LSECs increases CXCR4 expression, promoting TGF-β secretion and fostering a pro-fibrotic environment (Ding et al., 2014). However, on the fourth day after liver resection, restored Ang2 expression in LSECs further facilitates the formation of a pro-angiogenic microenvironment by enhancing vascular endothelial growth factor receptor-2 (VEGFR-2) expression (Hu et al., 2014). Therefore, investigating the molecular mechanisms regulating CXCR4 and CXCR7 expression could provide new therapeutic targets for promoting liver regeneration (Figure 3).

After liver resection, VEGF secreted by hepatocytes induces the expression of stromal cell-derived factor 1 (SDF1), which recruits BM CXCR7 (+) progenitors of liver sinusoidal endothelial cells (sprocs) to the hepatic sinusoids, where they differentiate into LSECs, aiding liver regeneration and repair (DeLeve et al., 2016). In contrast, CCl_4_-induced acute liver injury upregulates hypoxia-inducible factor-1α (HIF-1α), leading to increased CXCR4 expression in LSECs. This elevates platelet-derived growth factor-BB (PDGF-BB) expression and receptor activation, which inhibits CXCR7 expression and suppresses hepatocyte regeneration. Additionally, PDGF-BB secreted by LSECs activates adjacent HSCs, promoting a pro-fibrotic microenvironment (Fang et al., 2023). Thus, PDGF receptor inhibitors may serve as potential therapeutic agents to promote CXCR7 expression and enhance liver regeneration. These findings suggest therapeutic targets for enhancing CXCR7 expression in LSECs, promoting HGF and Wnt-2 secretion, and creating a pro-regenerative vascular microenvironment (Figure 3).

#### 3.1.2 Bone marrow vascular microenvironment

Growth factors, signaling molecules, exosomes, and functional regulatory proteins secreted by VECs in the bone marrow vasculature (BMV) play a crucial role in regulating the self-renewal and homing activities of hematopoietic stem cells, significantly influencing their development (Chen J. et al., 2020; Ramalingam et al., 2021; Ho et al., 2022). The BMV controls the adhesion and expansion of hematopoietic stem cells through the Kruppel-like factor 6a (KLF6a) signaling pathway (Xue et al., 2017). Conditional knockdown of angiopoietin-like protein 2 (ANGPTL2) expression in VECs demonstrated that ANGPTL2 derived from VECs supports the regenerative capacity of hematopoietic stem cells, enabling BM vascular microenvironment to maintain hematopoietic stem cells stemness (Yu et al., 2022). In addition, Chen Y. et al. (2021) showed that aging induces the formation of a fibrotic hematopoietic microenvironment that inhibits regeneration, thus hindering the regeneration of old organs. However, vascular microenvironment deterioration, hematopoietic stem cells dysfunction, and regeneration defects can be ameliorated by blocking Interleukin-1 (IL-1) signaling in endothelial cells (ECs). Therefore, IL-1 is a key inflammatory mediator that can be used to ameliorate the aging hematopoietic microenvironment of the BM (Mitchell et al., 2023).

Thus, VECs in the vascular microenvironment can regulate parenchymal cell function by secreting various signaling molecules, affecting tissue and organ function. However, physicochemical factors in the vascular microenvironment can also have a variety of effects on VEC function.

### 3.2 Physiochemical interactions in the vascular microenvironment and ECs in cardiovascular diseases

The influence of physicochemical factors in the vascular microenvironment on VECs plays a crucial role in the development of cardiovascular diseases (CVDs). VECs are important sensors of blood shear stress, with physiological laminar shear stress promoting the maintenance of vascular homeostasis (Cui et al., 2017; Iring et al., 2019; Chandran Latha et al., 2021). In contrast, oscillatory shear stress (OSS) alters the expression and structure of cell–cell adhesion proteins involved in vascular permeability (Chiu and Chien, 2011) and triggers the release of inflammatory molecules from ECs, resulting in apoptosis and necrosis (Zhou Y. et al., 2019; Chandran Latha et al., 2021; Saberianpour et al., 2021; Dupuy et al., 2022). Moreover, activation of the endothelial Toll-like receptor (TLR4) has been shown to play a critical role in OSS-induced endothelial inflammation, potentially serving as a key initiator of AS development (Qu et al., 2020).

However, in addition to regulating the secretion of nitric oxide (NO) and vasoactive substances from VECs, high glucose synergize with OSS inhibiting the activities of phosphor endothelial nitric oxide synthase (p-eNOS), p-protein kinase B (AKT), and p-focal adhesion kinase in human aortic ECs, leading to a disturbance in vascular homeostasis and even causing or aggravating vascular remodeling and the occurrence of AS (Patibandla et al., 2014). Furthermore, a high-fat environment increases endothelial permeability to lipoproteins, leading to the accumulation of low-density lipoprotein in ECs (Jensen and Mehta, 2016; Akhmedov et al., 2021). This process induces the secretion of more vasoconstrictor factors, such as endothelin-1 (ET-1), while decreasing the release of vasodilator factors, primarily NO. In addition, the expression of leukocyte adhesion and migration molecules, such as vascular cell adhesion molecule (VCAM), intracellular adhesion molecule (ICAM) and monocyte chemotactic protein-1 (MCP-1), is also increased, triggering an inflammatory response leading to EC damage and apoptosis (Milutinović et al., 2019).

The vascular microenvironment also encompasses various cytokines and growth factors that influence the function of VECs. For instance, antiangiogenic factors, including ET-1 (Babaahmadi-Rezaei et al., 2022), c-reactive protein (CRP) (Aminuddin et al., 2020), and tumor necrosis factor-alpha (TNF-α) (Wang et al., 2021; Babaahmadi-Rezaei et al., 2022), play critical roles. Conversely, proangiogenic factors, including VEGF (Shoeibi et al., 2018), HIF-1α (Zhou J. et al., 2021), basic fibroblast growth factor (b FGF) (Shoeibi et al., 2018), and PDGF (Shoeibi et al., 2018), promote angiogenesis. Furthermore, Ang-1 (Khamchai et al., 2022) and Ang-2 (Huang B. et al., 2021) are involved in regulating vascular permeability.

### 3.3 Interacting effects of VECs with stem/progenitor cells

Zaniboni et al. (2015) reported that vascular progenitor cells from porcine aorta could differentiate into ECs and SMCs, suggesting the existence of common vascular progenitor cells for different cell types. Numerous studies have also shown that stem/progenitor cells, such as EPCs and MSCs, are the main components of the vascular microenvironment. These cells serve as progenitors for VECs and vascular mesenchymal stromal cells and act as an important reserve source for terminally differentiated cells (Wang et al., 2018; Van Nguyen et al., 2021; Zhou N. et al., 2021). In contrast, Shih et al. (2012) found that the vascular endothelial microenvironment is a key site for determining the fate of stem/progenitor cells, which can influence their biological properties and plasticity. For instance, selective activation of the Integrin β2 and Notch signaling pathways in ECs determines whether peripheral blood-derived EPCs differentiate into ECs or macrophages, a process that ultimately affects the repair of the damaged endothelium (Shih et al., 2012). Furthermore, the secretion of platelet-derived growth factor-D (PDGF-D) by ECs has been shown to promote the proliferation, migration, adhesion and tube-forming ability of EPCs, thus contributing to AS (Zhang et al., 2019). All the studies suggest that VECs interact with their surrounding stem/progenitor cells but with some tissue specificity. Therefore, we summarize the vascular microenvironment across different sites below.

#### 3.3.1 Bone marrow vascular microenvironment

The BM vascular microenvironment includes vascular cells and hematopoietic stem cells. The BM vascular microenvironment regulates the stemness and differentiation properties of hematopoietic stem cells and is involved in the development of hematopoietic diseases, including myelodysplastic syndromes (MDS) (Mosteo et al., 2021) and acute myeloid leukemia (AML) (Yamashita et al., 2020). Dysfunction of the BM vascular microenvironment is also a major cause of hematopoietic stem cells graft failure. For example, the increase in apoptosis of hematopoietic stem cells induced by sinusoidal ECs via the Fas and Caspase-3 pathways is the main mechanism causing disturbances in the BM vascular microenvironment (Kaufman et al., 2014), which greatly exacerbates the incidence of acute graft-versus-host disease.

Abnormal alterations of stem/progenitor cells in the BM vascular microenvironment have been implicated in the development of various hematological malignancies. In a study involving 56 patients with low-risk myelodysplastic syndromes (MDS), EPCs from these patients were found to exhibit altered methylation patterns in genes such as p15 inhibitor of cyclin-dependent kinase 4b (p15^INK4b^), death-associated protein kinase (DAPK1), cadherin 1 (CDH1), or suppressor of cytokine signaling 1 (SOCS1), which triggered the abnormal expression of Wnt signaling-related miRNAs, ultimately leading to defective differentiation marker expression in EPCs and accelerates the progression of MDS (Teofili et al., 2015). However, ECs in the vascular endothelial microenvironment may become a new direction for modulating the state of the BM vascular microenvironment and treating hematological malignancies. For the first time, it has been shown that, small extracellular vesicles from T-ALL leukemia cells remodel the vascular microenvironment and suppress normal hematopoiesis by activating the protein kinase R-like endoplasmic reticulum kinase (PERK)/eukaryotic initiation factor 2 (eIF2)/activating transcription factor 4 (ATF4)/jagged1 (JAG1) axis in ECs. Conversely, targeting endothelial PERK can restore vascular microenvironment function, induce leukemia cell apoptosis, and increase residual hematopoietic progenitor cells, providing a potential therapeutic strategy to improve T-ALL treatment (Liu et al., 2022).

#### 3.3.2 Tumor vascular microenvironment

The vascular microenvironment demonstrates remarkable plasticity, maintaining normal cellular functions under physiological conditions. However, under pathological conditions, cancer cells exploit paracrine signaling to induce gene and metabolic reprogramming in ECs, reshaping the vascular microenvironment into a tumor vascular microenvironment that facilitates tumor growth, metastasis, angiogenesis, and immune evasion (Cleveland and Fan, 2024). For instance, in non-small cell lung cancer, cancer cells suppress the expression of the transcriptional regulator forkhead box protein 1, reprogramming lung ECs into tumor-associated endothelial cells (TECs) that form leaky blood vessels, which promote tumor growth and metastasis (Bian et al., 2024). Additionally, targeted knockout of p21-activated kinase 4 (PAK4) reprograms the transcriptome of tumor ECs through specific mechanisms, reduces vascular permeability, and reduces T cell adhesion to ECs, thus providing a novel therapeutic strategy to improve tumor vascular microenvironment and enhance immunotherapy efficacy (Ma et al., 2021). Furthermore, tumor cells can enhance their invasion and metastasis by promoting neoangiogenesis, lacking basement membrane structures, leading to cancer progression (Yip et al., 2021).

Tumor cells at the primary site can also reach the metastatic site through the circulation by releasing a variety of secreted factors, including tumor-secreted factors, and extracellular vesicles, thereby affecting and remodeling the vascular microenvironment at the metastatic site and forming the pre-metastatic microenvironment (PMN) of the tumor (Peinado et al., 2017). The PMN is characterized by endothelial permeability, which facilitates the invasion of tumor cells into surrounding tissues (Fan et al., 2022). Some studies have suggested that inhibition of the synthesis of cyclooxygenase-1 (COX-1) or thromboxane A2 (TXA2) can prevent the formation of the PMN (Lucotti et al., 2019), which may be a new direction to inhibit cancer metastasis.

#### 3.3.3 The vascular microenvironment of the nervous system

In the adult central nervous system, signals from ECs regulate the proliferation and differentiation of neural stem cells and promote the migration of neural progenitors and immature neurons to the site of nerve injury (Rivera et al., 2017). Studies have shown that activated forkhead box C1 promotes the proliferation and self-renewal of arachnoid-soft meningeal stem cells by restoring the neurovascular endothelial microenvironment in a cerebral ischemia/reperfusion model (Lei et al., 2022). Furthermore, structural and functional aging of the vascular endothelial microenvironment is a primary factor underlying the decline in brain plasticity and repair capacity (Rojas-Vázquez et al., 2021). Therefore, regulating the function of ECs in the vascular endothelial microenvironment of the nervous system may be a new approach to promoting neural stem cell proliferation and neuronal regeneration (Rivera et al., 2017).

## 4 Vascular wall microenvironment and associated stem cells

### 4.1 Concept of the vascular wall microenvironment

The traditional view is that the tunica adventitia is merely a structure in which fibrous material solidifies. AS, the basic pathological process of several CVDs, begins with endothelial damage, which leads to inflammatory cell infiltration and lipid deposition. The entire pathological process of AS is believed to occur from the inside out in the vessel wall (Maiellaro and Taylor, 2007; Majesky et al., 2011; Luo et al., 2021). In contrast to this traditional view, some scientists have suggested that the tunica adventitia is the initial responder and activator of the vascular response to injury (Tinajero and Gotlieb, 2020). Fibroblasts within the tunica adventitia have been identified as key contributors to the development of AS. Following vascular injury, fibroblasts generate significant amounts of NAD(P)H oxidase-derived reactive oxygen species, which promote SMC hypertrophy and neointimal hyperplasia (Meijles and Pagano, 2016). Furthermore, exosomes secreted by fibroblasts deliver miR-21-5p to VSMCs to promote vascular calcification (Zheng et al., 2023). These pathways can lead to or exacerbate atherosclerotic plaque formation and vascular sclerosis, contributing to the development of CVD. Furthermore, resident macrophages and T cells have been found to be present in the tunica adventitia (Sedding et al., 2018); therefore, researchers are increasingly speculating that the development of vascular inflammation in AS is closely related to the tunica adventitia. We emphasize the importance of the vascular wall microenvironment within the overall vascular microenvironment.

An increasing number of studies have shown that the VV contains a large number of stem/progenitor cells, known as the stem cell niche or angiogenic zone (Zengin et al., 2006). Importantly, progenitor cells for VECs, SMCs, and perivascular cells are not exclusively derived from the BM; a substantial portion originates from stem cell niches within the VV (Chambers et al., 2021). Thus, stem cell niches may serve as a reservoir of vascular cells.

In summary, we propose that the microenvironment of the vascular wall is composed of connective tissue, fibroblasts, and the VV in the tunica adventitia and highlight the role of potential stem cell niches in the VV in vascular regulation (Siow and Churchman, 2007).

### 4.2 Role of the VV in the vascular wall microenvironment

#### 4.2.1 Structure and function of the VV

The VV, first discovered over 150 years ago, is a dynamic microvascular system situated between the tunica adventitia and the tunica media. Its primary function is to supply nutrients to the mesothelial two-thirds of the vessel wall. The VV consists of ECs and SMCs in a regular laminar arrangement (Xu et al., 2015). The first-order VV (>100 μm), which may also have a connective tissue layer similar to that of large vessels, runs longitudinally along the vessel and branches into second-order VV (<100 μm), which penetrate the tunica adventitia and extend into the tunica media in a perpendicular direction (Billaud et al., 2017; Phillippi, 2022). Only a small proportion of secondary trophoblast vessels infiltrate the epithelial or intima-media layers. Studies have revealed that secondary trophoblast vessels are rare in the vasculature of healthy adults but are abundantly present in the vasculature of individuals with AS (Barger et al., 1984). This observation suggests the potential involvement of the VV in the development of AS, although the specific mechanisms remain unclear.

Studies have shown that the VV is not only an important channel for the migration of inflammatory cells into the intima, such as macrophages and leukocytes but also a pathway for the transport and mobilization of stem/progenitor cells from the vessel wall into the intima. Immunofluorescence staining and PKH26-labeled macrophage injection experiments demonstrated that circulating macrophages primarily accumulate in the VV of injured arteries. After entering the VV, these macrophages further infiltrate the tunica media and neointima (Li et al., 2020). Thus, the VV may contribute to vascular inflammation by recruiting and transporting macrophages. Moreover, studies have reported that 7 and 14 days after arterial injury, β-galactosidase-labeled adventitial cells were observed in the tunica media and intima, respectively. These adventitial cells contain various stem cell markers (Mallawaarachchi et al., 2005). When transplanting Sca-1-positive stem cells derived from the adventitia into the adventitia of venous grafts in ApoE^−/−^ mice, Sca-1-positive stem cells were detected in AS lesions in the intima (Hu et al., 2004). These studies have demonstrated that the VV also serves as a pathway for transporting stem/progenitor cells from the adventitia to the intima. Interestingly, the adventitial VV recruit inflammatory cells, inducing the production of matrix metalloproteinase-9 (MMP-9) and SDF-1, which can mobilize adventitial stem cells. These stem cells may migrate to the intima via the VV and accumulate there (Hu and Xu, 2011). These studies further illustrate that the VV functions as a crucial pathway, enabling the migration of inflammatory cells, such as macrophages and leukocytes, into the intima, while also acting as a conduit for the transport and mobilization of stem/progenitor cells from the vessel wall to the intima. Subsequently, the stem/progenitor cells entering the intima can differentiate into SMCs, leading to intimal thickening and accelerated plaque growth (Mulligan-Kehoe and Simons, 2014). Moreover, following vascular injury, activated fibroblasts can differentiate into myofibroblasts, driving VV proliferation and macrophage infiltration by promoting VEGF secretion and activating the VEGFR2/ERK1/2 signaling pathway (Li et al., 2020). Therefore, the VV may be an important structure involved in the pathological process of AS (Majesky et al., 2011; Upcin et al., 2021).

#### 4.2.2 Stem/progenitor cells in the VV angiogenic zone

In 2004, Hu et al. (2014) reported the presence of a stem/progenitor cell niche in the outer membrane of the aortic root in adult ApoE^−/−^ mice. They demonstrated that stem/progenitor cells from this site can differentiate into SMCs. Furthermore, Pasquinelli et al. (2009) identified a significant population of CD34^+^ stem/progenitor cells located between the tunica media and tunica adventitia of the human thoracic aorta and femoral arteries. Billaud et al. (2017) further isolated and characterized cells from the stem/progenitor cell niche in the human thoracic aorta, revealing that EPCs and MSCs are key components of this niche (Huang C. et al., 2021; Chen et al., 2022). More recently, a growing number of stem/progenitor cells have been isolated from the VV region of the tunica adventitia in large blood vessels, including the human pulmonary artery, adult ascending aorta, and internal thoracic artery (Majesky et al., 2011; Upcin et al., 2021). These findings suggest the presence of stem/progenitor cell niches within the VV region of the vascular wall. Stem/progenitor cell niches in the VV region contain cells capable of differentiating into ECs, hematopoietic cells, and local immune cells, leading to their designation as the “angiogenic zone” (Zengin et al., 2006).

Stem/progenitor cell niches are pivotal in regulating vascular function and may also play a role in the vascular inflammatory processes associated with AS. Evidence suggests that under pathological conditions, inflammatory cells in the intima are more likely to originate from the “angiogenic zone” of the VV rather than the circulating vasculature (Maiellaro and Taylor, 2007; Sedding et al., 2018; Li et al., 2021). Moreover, Billaud et al. (2017) have demonstrated that the stem/progenitor cell niche of the adult thoracic aorta may be a pivotal factor in the pathological remodeling of the aortic wall. Therefore, it can be concluded that the stem/progenitor cell niche in the VV region not only responds to vascular injury but is also an important site for vascular immune surveillance and inflammatory responses (Toledo-Flores et al., 2019; Owusu and Barrett, 2021). In addition, it has been demonstrated that transplantation of stem/progenitor cells from the tunica adventitia stem/progenitor cell niche to the site of ischemia can stimulate the production of ECs and the formation of the cardiovascular system. For example, progenitor cells transplanted from the aortic periphery of mice into an ischemic hindlimb model facilitated ECs formation and neovascularization, increasing perfusion by up to 50% compared to controls (Toledo-Flores et al., 2019). These findings suggest that stem/progenitor cells derived from such niches represent a promising avenue for research and therapeutic applications in ischemic injury repair.

Concurrently, stem/progenitor cells are influenced by the vascular microenvironment, which in turn affects vascular function and the regulation of the vascular wall microenvironment. The function of stem/progenitor cells can be affected by various factors within the vascular microenvironment, including peripheral nerve tissue, lymphoid tissue, cytokines, and the paracrine effects of PVAT (Ma et al., 2022). Furthermore, it has been demonstrated that the interaction between EPCs and MSCs within the stem/progenitor cell niche can be enhanced by angiogenic factors, such as VEGF, or exosomes (Liu et al., 2021; Wu et al., 2021; Zhang et al., 2021). In conclusion, the stem/progenitor cell niche in the VV region, also known as the “vascular niche”, contains stem/progenitor cells that may be involved in pathological processes such as vascular inflammatory responses and pathological remodeling of the vasculature, which are crucial for the development of CVDs.

#### 4.2.3 The VV is involved in the pathological process of CVD

It has been demonstrated that modifications to the vascular wall microenvironment, particularly those centered on VV, can directly or indirectly influence vascular endothelial function and homeostasis, thereby contributing to the pathogenesis of various vascular diseases (Li et al., 2020; Chambers et al., 2021; Farias-Itao et al., 2022; Sano et al., 2022). AS, restenosis, diabetes mellitus, and hypercholesterolemia are all associated with an increased VV of the diseased perivascular membrane (Luo et al., 2021). However, the high permeability of the nascent VV endothelium to lipoproteins and leukocytes results in the formation of plaques and intraplate hemorrhage and plaque rupture (Yan and Gotlieb, 2023). Anti-VV angiogenesis treatments have shown promise in mitigating these pathological processes. For instance, studies have demonstrated that inhibiting VV angiogenesis reduces atherosclerotic plaque formation and vascular remodeling in hypercholesterolemic mice (Bogdanov et al., 2022). Similarly, using soluble VEGFR-1 and VEGFR-2 to inhibit VV neovascularization in the rabbit aortic perithelium significantly reduced the number and extent of VVs, suggesting a potential therapeutic strategy to mitigate in-stent restenosis (de Vries et al., 2018). This approach may serve as a novel strategy to mitigate in-stent restenosis.

The VV is closely associated with the inflammatory response in the pathological process of AS and has gradually become a prominent area of research in this field. Upon entering the circulatory system, fluorescently labeled macrophages have been shown to initially undergo rolling, adhesion, and migration within the vasculature, and subsequently localize in the tunica adventitia (Li et al., 2020). Furthermore, elevated expression of VEC adhesion molecules and selectin molecules, such as VCAM-1 and P-selectin, indicates that the VV may act as a novel site for inflammatory cell chemotaxis, homing, and activation (Shimosawa et al., 2019). Consequently, it is postulated that the VV represents a pivotal site for the vascular inflammatory response. Nevertheless, the underlying pathological mechanism that initiates VV neovascularization and inflammatory infiltration remains unclear.

**FIGURE 4:**
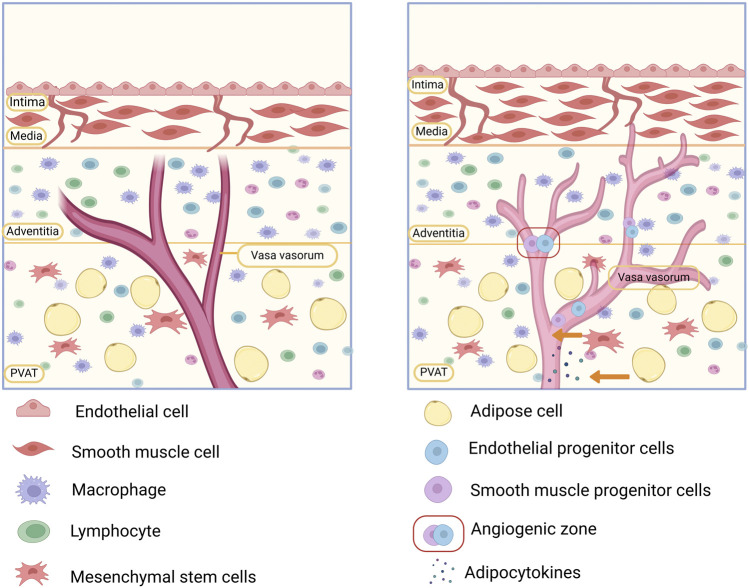
Involvement of the vascular microenvironment in the development of atherosclerosis (AS). The vasa vasorum (VV) in the vascular microenvironment is a conduit for the delivery of macrophages and inflammatory factors. Cytokines secreted by fibroblasts promote VV expansion and neogenesis, while the high permeability of the neovascularized VV can lead to lipid deposition and plaque formation. In addition, stem progenitor cells in the “angiogenic zone” of the VV and perivascular adipose tissue (PVAT) can differentiate into smooth muscle cells (SMCs), which are involved in vascular remodeling, both of which may lead to the development of AS.

In conclusion, the VV within the vascular wall microenvironment plays a critical role in macrophage recruitment, neointimal lesion formation, and vascular remodeling. Its contribution to the development of AS underscores its close association with CVDs. Thus, targeting VV neovascularization or blocking its role in inflammatory cell transmission could offer a novel therapeutic approach to halt the progression of AS (Figure 4).

## 5 Perivascular microenvironment and associated stem cells

### 5.1 PVAT represents the perivascular microenvironment

In addition to the intima, media, and adventitia of the vessel wall, an additional layer of adipose tissue, known as PVAT, is situated externally to the vessel wall. PVAT is composed of adipocytes, MSCs, and extracellular matrix. It has been proposed that PVAT functions as a unique endocrine or paracrine organ (Angueira et al., 2021). Cytokines released by cells within PVAT may reach the vascular endothelium through endocrine or paracrine mechanisms or via the VV(Man et al., 2022), thereby influencing EC function (Brown et al., 2014; Tanaka and Sata, 2018; Kubrova et al., 2020). The effects of cytokines released by PVAT have a dual nature. Under physiological conditions, PVAT dynamically regulates vascular tone by releasing vasodilatory and vasoconstrictive factors, thereby maintaining normal vascular function (Zierold et al., 2021). PVAT produces various bioactive factors, including NO and lipocalin, which promote relaxation in VSMCs (Man et al., 2020). Conversely, under pathological conditions, damaged PVAT secretes bioactive factors that adversely affect vascular elasticity, potentially leading to hypertension (Mu et al., 2022). Thus, while PVAT supports vascular microenvironment stability and normal vascular physiology under healthy conditions, PVAT dysfunction initiates vascular pathological events (Katsiki and Mikhailidis, 2021). Obesity has been shown to induce dysfunction in PVAT and exacerbate vascular remodeling. This process is mediated by activation of the nod-like receptor pyrin domain containing 3/IL-1 signaling pathway, which promotes the proliferation and differentiation of adventitial fibroblasts within the vascular microenvironment, thereby driving pathological vascular remodeling (Zhu et al., 2019). These findings suggest that PVAT may serve as a potential therapeutic target to mitigate the risk of CVD (Fleenor et al., 2022). Therefore, we categorize PVAT as part of the perivascular microenvironment. The perivascular microenvironment does not exist as an isolated entity but exerts a synergistic influence on vascular stability by regulating both the endothelial and vessel wall microenvironment. For example, PVAT dysfunction has been associated with the formation of unstable plaques through the promotion of VV dilation and neovascularization (Tinajero and Gotlieb, 2020; Yan and Gotlieb, 2023). Moreover, multigene analysis studies of human abdominal aortic aneurysms (AAAs) have shown that PVAT is closely associated with autoimmune and inflammatory responses in AAAs (Piacentini et al., 2019). An AAA induces the upregulation of proinflammatory genes, such as protein tyrosine phosphatase receptor type C (PTPRC), C-X-C motif chemokine ligand 8 (CXCL8), lymphocyte-specific protein tyrosine kinase (LCK), C-C motif chemokine ligand 5 (CCL5), and MMP-9, while suppressing the expression of the anti-inflammatory gene peroxisome proliferator-activated receptor gamma (PPARγ), thereby contributing to inflammation in the adjacent aortic wall and playing a role in the pathophysiology of AAA (Meekel et al., 2021). Additionally, PDGF-D derived from PVAT has been shown to promote epithelial fibroblast proliferation, migration, and the expression of inflammatory cytokines, playing a critical role in AAA formation during obesity (Zhang et al., 2018). Therefore, the release of inflammatory mediators and immune regulation within PVAT may provide novel therapeutic targets for the treatment of AAA. Nevertheless, the precise roles and mechanisms through which PVAT influences these pathological processes remain poorly understood. Further research is required to elucidate its involvement and therapeutic potential.

### 5.2 Stem cells in PVAT

PVAT is a rich source of MSCs, which possess the capacity to differentiate into osteoblasts, adipocytes, and chondrocytes (Ye et al., 2021). Stem/progenitor cells, including those derived from PVAT, can differentiate into perivascular cells that contribute to the formation of the VV (Angueira et al., 2021; Asano et al., 2021). However, excessive differentiation of these cells into adipocytes is a primary mechanism underlying adipose tissue dysfunction (Scott et al., 2019). PVAT dysfunction plays a critical role in the progression of AS by influencing the behavior of stem/progenitor cells. Dysfunctional PVAT releases proinflammatory factors and free fatty acids, which induce the differentiation of PVAT-resident stem cells into VSMCs. This process promotes vascular remodeling and accelerates the development of AS (Klöting and Blüher, 2014; Ahmadieh et al., 2020). In addition, stem/progenitor cells in the perivascular microenvironment modulate the cellular activity of stem/progenitor cell niches in the vascular wall microenvironment, leading to further deterioration of AS by promoting the formation of hyperpermeable neovascular VV. For instance, transplantation of mouse PVAT into endothelium-injured common carotid arteries resulted in the production of CRP by injured ECs. CRP subsequently stimulated VEGF production by PVAT-derived stem cells, which promoted neovasculogenesis and contributed to the deterioration of vascular disease (Manka et al., 2014; Chen J.-Y. et al., 2020).

Conversely, ECs within the vascular endothelial microenvironment also regulate the function of PVAT. It was demonstrated that the function of PVAT could be regulated in an inverse manner through the targeted intervention of VECs. For example, Peterson et al. showed that disrupting the expression of the heme oxygenase-1 (HO-1) gene in VECs successfully inhibited the differentiation of PVAT-derived MSCs into adipocytes. This intervention enhanced PVAT functionality, indicating that HO-1 may act as a critical mediator in maintaining PVAT and vascular microenvironment homeostasis (Peterson et al., 2019).

In conclusion, PVAT is a rich source of stem and progenitor cells, particularly in the presence of MSCs. MSCs can serve as a potent reservoir for vascular cells, whereas their differentiation into SMCs and promotion of VV neovascularization in pathological states accelerate the progression of AS. Second, the secretion of cytokines by adipocytes in PVAT can affect the function of stem/progenitor cells through the paracrine pathway. Moreover, the function of stem/progenitor cells within the microenvironment of the vessel wall can be influenced by the VV pathway, which leads to the differentiation of SMCs and vascular remodeling. Therefore, regulating the function of PVAT adipocytes may offer a novel target to inhibit atherosclerosis progression, but further investigation is needed to clarify the mechanisms of interaction within the vascular microenvironment (Figure 4).

## 6 Discussion

The vascular microenvironment is essential for tissue homeostasis and disease regulation, with its complex structure and molecular network influencing both healthy and pathological states. Studying its regulatory mechanisms provides insights into disease development and guides the exploration of potential therapeutic strategies for various conditions.

In the vascular endothelial microenvironment, the overexpression of vascular endothelial HGF and the inhibition of the profibrotic gene NOX4 contribute to the development of a profibrotic vascular environment, which impacts liver and lung regeneration. This offers a new perspective on the treatment of fibrotic diseases (Cao et al., 2017). However, the inhibition of DGKG expression in hepatic ECs has been shown to reduce TGF-β1 secretion in the liver, thereby decreasing angiogenesis and immune evasion in hepatocellular carcinoma (HCC). Consequently, this leads to delayed tumor progression and improved survival (Zhang et al., 2024). Additionally, in the BM vascular microenvironment of AML, inhibiting the secretion of IL-4 by ECs promotes megakaryocyte proliferation, improves platelet count, and enhances the effectiveness of chemotherapy (Gao et al., 2019). Therefore, a thorough investigation of the molecular network and interaction mechanisms within the vascular microenvironment, with a particular focus on ECs, may uncover precise targets for the treatment of a wide range of diseases.

Similarly, interventions targeting the vessel wall and perivascular microenvironment may contribute to disease regression. For instance, various antiangiogenic factors, such as thalidomide, endostatin, angiostatin, and recombinant plasminogen activator inhibitor-1_23_ (rPAI-1_23_), can inhibit neoangiogenesis of VV, thereby alleviating AS lesion progression (Boyle et al., 2017). It was found that mechanical damage to the endothelium, induced by stent implantation, leads to the secretion of VEGF, thereby promoting angiogenesis, which is a significant cause of stent restenosis (Sluimer et al., 2008). Hytönen et al. injected adenoviruses encoding soluble VEGF receptors 1 (sVEGFR1), and 2 (sVEGFR2) into local arteries via a catheter and implanted them in bare-metal stents at the same locations which was shown to inhibit VV neogenesis and effectively prevent in-stent restenosis (Hytönen et al., 2018).

PVAT in the perivascular microenvironment is a key target for CVDs therapy (Gollasch, 2017). For instance, PPARγ in PVAT adipocytes reduces inflammation and oxidative stress, improving the arterial microenvironment and exerting an anti-atherosclerotic effect (Chen J. Y. et al., 2021). This supports the use of PPARγ agonists, such as rosiglitazone and pioglitazone, in managing diabetes and obesity in atherosclerosis patients (Ryan et al., 2007; Powell et al., 2012). Notably, transplantation of thoracic PVAT into the abdominal aorta altered abdominal PVAT, inhibiting macrophage infiltration and MMP-9 production, while preventing VSMC apoptosis by promoting adipocyte cartilage oligomeric matrix protein release, ultimately reducing AAA formation (Huang et al., 2023). Additionally, PVAT holds potential as a biomarker for diagnosing and assessing vascular function, aiding in the prevention of CVDs (Antoniades et al., 2023).

In summary, research on the vascular microenvironment has provided valuable insights into disease mechanisms and opened new avenues for precision treatments of fibrosis, tumors, and CVDs. Despite significant progress, further investigation is needed to understand stem cell activity in this context and its therapeutic potential. Future integration of advanced imaging, machine learning, and molecular biology will deepen our understanding of vascular microenvironment, offering innovative strategies for disease diagnosis, treatment, and prognosis (Goudot et al., 2024).
